# Environmental Determinants of Bronchial Asthma among Saudi School Children in Southwestern Saudi Arabia

**DOI:** 10.3390/ijerph14010022

**Published:** 2016-12-28

**Authors:** Jobran M. Alqahtani, Ahmed M. Asaad, Nabil J. Awadalla, Ahmed A. Mahfouz

**Affiliations:** 1 Department of Pediatrics, College of Medicine, Najran University, Najran 1988, Saudi Arabia; jobrancv@yahoo.com; 2 Department of Clinical Microbiology, College of Medicine, Najran University, Najran 1988, Saudi Arabia; ahmedmoradasaad@hotmail.com; 3 Department of Family and Community Medicine, College of Medicine, King Khalid University, Abha 61421, Saudi Arabia; njgirgis@yahoo.co.uk; 4 Department of Community Medicine, College of Medicine, Mansoura University, Mansoura 35516, Egypt; 5 Department of Epidemiology, High Institute of Public Health, Alexandria University, Alexandria 21511, Egypt

**Keywords:** school children asthma, environmental, dietary factors, Southwestern Saudi Arabia

## Abstract

The aim here was to study the possible environmental and dietary determinants of asthma among school-aged children in Southwestern Saudi Arabia. In a cross-sectional study on a representative sample in Najran in Southwestern Saudi Arabia using an Arabic version of the modified ISAAC Phase III, parent-administered questionnaire data were collected. Skin prick tests (SPTs) were performed. The study included 1700 school children, out of them 468 (27.5%) were diagnosed with, cases of bronchial asthma and 20.8% (353) reported a 12-month nocturnal cough (as a proxy of severe asthma). In multivariable analysis, the study identified the following risk factors for having asthma or severe asthma: having dogs in the house, being male, being exposed to dense truck traffic on the street, using wood as a cooking fuel, conducting vigorous exercise, consuming eggs, consuming vegetables, having an allergic sensitization to dog hair, and being exposed to *Cladosporium*, pigweed, and Bermuda grass. On the other hand, the following food stuffs were found to be protective: seafood, fruit, and dairy products. Comprehensive school educational programs for both children and their parents should be adopted to prevent the use of wood in cooking and heating, to ensure that house pets are properly cared for, and to encourage proper dietary habits. Physicians should be informed of the patterns of allergens in order to improve asthma diagnosis and management.

## 1. Introduction

Bronchial asthma is one of the most common chronic diseases among school children. It has significant public health influences, with impacts on patients’ quality of life, healthcare expenses, morbidity, and mortality [1,2]. The prevalence of schoolchildren asthma/wheeze is rising around the world and in Saudi Arabia [3,4,5,6].

The causes of the evolving epidemic are not entirely understood. Some suggestions are related to changes in lifestyle and environmental exposures [7,8] that trigger the immune system in early stages of life [9]. There is some evidence of an association between environment factors such as air pollution [10], indoor and outdoor allergens, and pollutants and the initiation and aggravation of asthma [1,11,12]. A number of studies have shown that some dietary factors could alter the risk of asthma [13], while others hypothesize that other food products could have a protective effect on allergies, such as fruits, vegetables [14], and oily fish [15]. On the other hand, protein-rich and fat-rich foods of animal origin could increase the risk of asthma [16].

Studies addressing the link between environmental and dietary factors and bronchial asthma in Southwestern Saudi Arabia are so far scarce and even lacking. The Najran region is located in the southwest of Saudi Arabia along the neighboring border of Yemen. It has an area of 360,000 km^2^. Updated data from the Ministry of Education showed that more than 111,000 male and female students are currently enrolled in Najran’s schools (primary, intermediate, and secondary levels).

Knowledge of the association between modifiable environmental risk factors and bronchial asthma in Southwestern Saudi Arabia can help the health authorities to plan and implement preventive strategies to reduce the disease burden in this area. Therefore, the aim of this study was to study the associations of possible environmental and dietary determinants of asthma among school-aged children in Southwestern Saudi Arabia.

## 2. Materials and Methods

Parents of all study subjects gave informed written consent for inclusion before the participation in the study. The study was conducted in accordance with the Declaration of Helsinki, and the protocol was approved by the Ethics and Research Committee of the College of Medicine, Najran University (No. 3642, date 15/12/2015).

### 2.1. Study Design and Target Population

The study is a cross-sectional study on a representative sample of male and female Saudi school children in Najran region, Southwestern Saudi Arabia.

### 2.2. Sample Size Determination and Sampling

Using the WHO manual for Sample Size Determination in Health Studies [17], at 95% confidence interval with a conservative estimate of the anticipated population proportion of 15% [18] and with an absolute precision of 2%, the minimal sample size required for the study was calculated to be 1537 students. To avoid loss of cases, a total sample of 1700 students (boys and girls) was planned to be included in the present study.

A two-stage stratified random sample of 1700 children was selected out of the primary, intermediate, and secondary schools. At the first sampling stage, the schools were classified into 2 groups according to geographical location and socio-economic level. A total of 20 schools and a total of 86 classes were identified in the sample.

### 2.3. Questionnaire Interview

A structured questionnaire was distributed among the study sample to be completed by their parents. The questionnaire is an Arabic validated version of the modified ISAAC Phase III parent-administered questionnaire [18,19]. Two questions only from the Bronchial Asthma (BA) module were included to identify physician-diagnosed asthma—“Have you ever had bronchial asthma at any time in the past?”—and nocturnal cough (as a proxy of severe asthma)—“In the last 12 months, have you had a dry cough at night, apart from a cough associated with a cold or chest infection?” The questionnaire also included the following information: demographic data such as age, sex, family pets (cat and dogs), the presence of smokers in the family, exposure to outdoor air pollution through the frequency of truck traffic on the street where children lived, the use of wood or coal as cooking fuel, the consumption of different food types, and other risk factors (vigorous exercise and paracetamol).

### 2.4. Skin Prick Tests

The skin prick tests (SPTs) were performed, using a panel of standardized allergenic extracts and the Stallerpoint device (Stallergenes, Paris, France). The allergens panel included *D. pteronyssinus* and *D. farina* (house dust mites), Berumda grass (*Cynodondactylon*) and Timothy grass (*Phleum pretense*) (grass pollens), ragweed (*Ambrosia*), mugwort (*Artimisia*), pigweed (*Amaranthus*), *Chenopodium album* (Lamb’s quarter) (weed pollens), *Candida albicans* (fungi), *Cladosporium* (molds), cat fur, dog hair, and horse hair (animal dander). Histamine hypochloride (10 mg/mL) and normal saline (0.9% NaCl solution) were used as positive and negative controls, respectively.

To determine the size of the wheals, their longest and perpendicular diameters were measured using a transparent ruler. The measurements were summed and then divided by 2 in order to obtain an average wheal diameter [20]. A positive SPT result was considered when the wheal diameter was >3 mm to at least one of the allergens or 3 mm larger than the negative control. The wheal diameter of positive and negative controls should be ≥3 mm and <3 mm, respectively [20].

### 2.5. Statistical Analysis

Data were coded, validated, and analyzed using SPSS PC+ version 22 software package. Frequency, percentage, arithmetic mean, median, and mode were used to present the data. Crude odds ratios (cORs), binary logistic multivariable analysis, adjusted odds ratios (aORs), and antecedent 95% confidence intervals (95% CIs) were used to identify potential risk factors. The binary logistic model was used to estimate the probability of a binary response. The dependent variables were having diagnosed asthma or having severe asthma as indicated by the core ISAAC questionnaire for asthma. The independent variables included in the present study were 10 variables covering personal and environmental factors, 10 dietary factors, and 13 allergens sensitization factors. Logistic regression measures the relationship between the dependent variables and the independent variables by estimating probabilities using a logistic function, which is a cumulative logistic distribution.

## 3. Results

### 3.1. Description of the Study Sample

The present study included 1700 school aged children. The sample included 851 (50.1%) boys and 849 (49.9%) girls. The majority of the study sample (990, 58.2%) were from primary schools, followed by intermediate (413, 24.3%) and secondary schools (297, 17.5%). The age of school children ranged from 7 years to 19 years with an average of 12.21 ± 3.34 years and a median of 12 years.

### 3.2. Prevalence of Asthma and Severe Asthma

Based on responses to core questions of ISAAC questionnaire for bronchial asthma the study showed that 468 (27.5%) school children were diagnosed with cases of bronchial asthma. The reported 12-month nocturnal cough rate was 20.8% (353). This figure can be considered as a proxy of severe asthma among the study children.

### 3.3. Determinants of Asthma and Severe Asthma

The study focused mainly on determinants of asthma (468) and severe asthma (353). The group without asthma were children free from any bronchial asthma responses (1096).

#### 3.3.1. Personal and Environmental Outdoor and Indoor Pollution Factors

After adjusting for other potential risk factors (using logistic binary multivariable analysis), the study showed (Table 1) that having dogs in the house (aOR = 2.78, 95% CI = 1.79–4.31) was a significant risk factor in having diagnosed asthma among school children in Najran. Similarly, the following risk factors were found for diagnosed asthma: being male, being exposed to dense truck traffic on the street where they lived, using wood as a cooking fuel, and performing vigorous exercise.

The multivariable analysis showed that dense truck traffic on the street where children lived (aOR = 2.13, 95% CI = 1.48–3.06) was a significant risk factor to having severe asthma (as reported by having a 12-month nocturnal cough) among school children in Najran. Similarly, the following risk factors were found for severe asthma; being male, having dogs, using wood as a cooking fuel, having a family member who smokes, and performing vigorous exercise.

#### 3.3.2. Dietary Factors

After adjusting for other potential risk factors (using multivariable analysis), the study showed (Table 2) that egg intake (aOR = 1.47, 95% CI = 1.11–1.95) and vegetable intake (aOR = 1.58, 95% CI = 1.18–2.11) were significant risk factors in having diagnosed asthma among school children in Najran. On the other hand, the following dietary factors were found to be protective for diagnosed asthma; seafood intake (aOR = 0.39, 95% CI = 0.17–0.88), fruit intake (aOR = 0.72, 95% CI = 0.54–0.96), and dairy product intake (aOR = 0.53, 95% CI = 0.40–0.71).

The multivariable analysis showed that egg intake (aOR = 1.60, 95% CI = 1.17–2.17) and vegetable intake (aOR = 2.17, 95% CI = 1.57–3.02) were also significant risk factors in having severe asthma among school children in Najran. On the other hand, the following dietary factors were found to be protective for severe asthma: seafood intake (aOR = 0.41, 95% CI = 0.16–0.95), fruit intake (aOR = 0.56, 95% CI = 0.40–0.97), and dairy product intake (aOR = 0.38, 95% CI = 0.28–0.53).

#### 3.3.3. Allergens Sensitization Factors

After adjusting for other potential risk factors (using multivariable analysis), the study showed (Table 3) that allergen sensitization to dog hair (aOR = 6.38, 95% CI = 1.80–22.53) was a significant risk factor in having a diagnosed asthma among school children in Najran. Similarly, allergen sensitization to *Cladosporium*, pigweed, and Bermuda grass were found to be significant risk factors.

The multivariable analysis showed that allergen sensitization to pigweed (aOR = 7.76, 95% CI = 1.79–33.56) and Bermuda grass (aOR = 2.55, 95% CI = 1.71–3.79) were also found to be significant risk factors in having severe asthma among school children in Najran.

## 4. Discussion

To our knowledge, this is the first study conducted in Southwestern Saudi Arabia that explores the environmental and dietary determinants of school-aged children diagnosed with asthma or severe asthma. The multivariable logistic regression revealed that the risk of diagnosed asthma or severe asthma were significantly higher among males, those with dogs, those that use wood or coal as cooking fuel, those exposed to dense neighborhood truck traffic, and those who perform vigorous exercise. Severe asthma was also significantly associated with having a family member who smokes.

Our results confirm previous studies in France [4] and Australia [21] which report that asthma is far more common in boys than girls in early childhood. This could be due to hormonal or biochemical differences by gender or due to different environmental exposures between boys and girls.

The current study revealed that children’s exposure to dogs was significantly associated with a higher risk of both asthma and nocturnal cough. Moreover, the SPT result regarding dog hair confirmed this finding. These findings are in accordance with previous studies in Bolivia [7] and Senegal [22]. The role of exposure and sensitization to dog’s allergens is unclear. This could be explained by the fact that increased risk of post-exposure bronchial hyper-responsiveness [12].

Using wood or coal as a cooking fuel was considered as a risk factor of asthma by several studies and positively associated with a degree of asthma symptoms as reported in Montreal, Canada [1], and Bolivia [7]. Indoor wood combustion is a major health problem in developing countries due to the associated higher risk of several respiratory morbidities, including bronchial asthma [23].

In the current study, outdoor air pollution was assessed by the density of truck traffic on the street where the children lived. The dense neighborhood truck traffic was positively associated with both diagnosed asthma and severe asthma. These results are consistent with those stated by ISAAC Phase III [24] as well as the prospective study conducted in Southern California showing a positive significant association between traffic-related air pollution and asthma [25]. This may be explained by the possible interaction of air pollutants, including ultrafine particles, metals, and polycyclic aromatic hydrocarbons and nitrogen oxides, with the respiratory tract and the immune system at a young age [7,25].

Doing vigorous exercise was found to be a significant predictor positively associated with physician diagnosed asthma or severe asthma by both univariate and multivariate analysis in the present study. There are controversial results regarding the effect of exercise on the development of asthma, some of which have described a relation between practicing higher levels of regular physical activity and a lower prevalence of asthma symptoms in Finland [26], whereas others have found that children who had wheezing were more physically active, as reported in The Netherlands [27].

The univariate and multivariate analysis in the current study revealed protective associations between seafood, fruit, and dairy product consumptions and diagnosed asthma and severe asthma. Moreover, positive associations were observed between vegetable and egg consumption and asthma. Our result of the protective effect of fish consumption with asthma is in agreement with the protective associations reported by other studies in children in The Netherlands [28] and globally in the ISAAC Phase II study [29]. Seafood is rich in long-chain n-3 polyunsaturated fatty acids, which have been shown to decrease the inflammatory responses and consequently minimize asthma symptoms [30]. Our current findings regarding the protective association of regular fruit intake and asthma are in line with the findings of the previous studies conducted in children reported globally in the ISAAC Phase II study [29] and a meta-analysis study [31]. Raw fruits are rich in antioxidants and other biologically active factors, which have been reported to minimize inflammatory responsiveness and asthma and improve pulmonary functions, as reported in ISAAC Phase II [29] and Phase III [32]. Our results reveal the presence of an inverse relationship between dairy intake and the risk of diagnosed and severe asthma. This was in line with a previous epidemiological study in Jeddah, Saudi Arabia [33]. Dairy foods are rich in saturated fatty acids, which have a mucosal protective effect [28].

The current study found a positive relation between vegetable consumption and the risk of asthma. This was in contrast to the findings of ISAAC Phase II [29] and Phase III [32], which observed a protective effect of regular vegetable consumption against asthma development. On the other hand, a lack of association between vegetable intake and asthma has been reported among Dutch children [28]. The possible explanation of this controversy could be the discrepancy in the selection of vegetables and the preparation methods.

The current study revealed positive associations between asthma and regular egg consumption. A significant association between allergies to egg and asthma has been shown in a previous study conducted in France [34], whereas other studies did not find such a significant association [32].

The results of allergen sensitization in the current study indicated a positive significant association between asthma and dog hair, mold fungi (*Cladosporium*), pigweed, and Bermuda grass pollens. On the other hand, house dust mites (HDMs) were not significantly associated with either asthma or severe asthma.

In contrast to the previous studies conducted in Boston, USA [35], and in Saudi Arabia [36], which found that indoor allergens, especially HDMs, were the most common allergens associated with asthma, our results detected a sensitization to dog and mold allergens *Cladosporium* fungi were the only significant indoor allergens positively associated with asthma. In New Mexico, USA, Ingram et al. [37] found that dogs and cats were the main allergens to which asthmatic children were sensitized in areas free of HDMs. *Cladosporium* is the most prevalent spore in temperate regions and is the most commonly identified indoor/outdoor fungus, and was found to be associated with seasonal and perennial allergy symptoms [12].

In line with our results, pigweed is defined as a major contributor to the aeroallergen load worldwide and has been shown to be a common aeroallergen inducing asthma [38]. Bermuda grass pollen was also found as a significant allergen that induces and exaggerates asthma severity [39]. The difference in the allergen pattern detected in the current study may reflect the unique environmental characteristic of the studied area. Najran is characterized by the presence of wide agricultural areas and exposed areas covered with grasses.

This study had some limitations. The questionnaire data may have been subject to recall bias and misclassification. However, the ISAAC questionnaire used in the study has been extensively validated in different populations. The specific IgE level was not performed for an objective assessment of allergic bronchial asthma. Nevertheless, the SPTs used in our study has proven to be a good procedure for detecting allergic asthma with other diagnostic techniques.

## 5. Conclusions

The present study identifies the significant modifiable environmental factors associated with diagnosed severe asthma in the Najran region in Southwestern Saudi Arabia: they include the use of wood or coal as cooking fuel, exposure to dense truck traffic, having household dogs, and having a family member who smokes. Moreover, the study demonstrated the protective effect of the frequent consumption of, e.g., seafood, fruit, and dairy products. Moreover, the pattern of allergens associated with asthma in the Najran region was recognized: these allergens include dogs, *Cladosporium* molds, pigweed, and Bermuda grass pollen. Prospective and experimental studies are needed to confirm these results. Comprehensive school-based educational programs for both children and their parents should be adopted to prevent the use of wood or coal in cooking and heating, to promote the cessation of smoking habits, to ensure that house pets are properly cared for, and to encourage proper dietary habits. Physicians should be informed of the pattern of allergens in order to improve correct asthma diagnoses and management.

## Figures and Tables

**Table 1:** Personal and environmental outdoor and indoor pollution factors among school children with diagnosed asthma, children with a 12-month nocturnal cough, and the no-asthma group.

Personal and Environmental Factors	No-Asthma Group ^1^ *N* = 1096	Ever (Diagnosed) Asthma *N* = 468	12-Month Nocturnal Cough *N* = 353
Age 13–20 in years: No (%)	471 (43.0%)	196 (41.9%)	138 (39.1%)
cOR ^2^ (95% CI)	-	0.96 (0.77–1.19)	0.85 (0.67–1.09)
aOR ^3^ (95% CI)	-	1.00 (0.79–1.27)	0.87 (0.66–1.14)
Sex: Male: No (%)	498 (45.4%)	275 (58.8%)	220 (62.3%)
cOR (95% CI)	-	**1.71 (1.37–2.13)**	**1.98 (1.55–2.54)**
aOR (95% CI)	-	**1.75 (1.35–2.27)**	**1.93 (1.45–2.58)**
Having dogs: No (%)	68 (6.2%)	53 (11.3%)	33 (9.3%)
cOR (95% CI)	-	**1.93 (1.32–2.81)**	**1.56 (1.01–2.40)**
aOR (95% CI)	-	**2.78 (1.79–4.31)**	**2.48 (1.50–4.10)**
Having cats: No (%)	478 (43.6%)	186 (39.7%)	132 (37.4%)
cOR (95% CI)	-	0.85 (0.68–1.06)	0.77 (0.60–0.98)
aOR (95% CI)	-	0.77 (0.60–1.00)	0.75 (0.56–1.01)
Dense truck traffic: No (%)	879 (80.2%)	417 (89.1%)	319 (90.4%)
cOR (95% CI)	-	**2.02 (1.45–2.80)**	**2.32 (1.58–3.40)**
aOR (95% CI)	-	**2.13 (1.48–3.06)**	**2.47 (1.61–3.80)**
Wood as cooking fuel: No (%)	11 (1.0%)	7 (1.5%)	7 (2.0%)
cOR (95% CI)	-	1.50 (0.57–3.88)	1.99 (0.77–5.18)
aOR (95% CI)	-	**3.01 (1.02–9.77)**	**4.16 (1.06–16.33)**
Wood as heating fuel: No (%)	124 (11.3%)	64 (13.7%)	37 (10.5%)
cOR (95% CI)	-	1.24 (0.89–1.72)	0.92 (0.62–1.35)
aOR (95% CI)	-	1.30 (0.90–1.89)	1.19 (0.77–1.84)
Family member smoking: No (%)	79 (7.2%)	48 (10.3%)	45 (12.7%)
cOR (95% CI)	-	1.47 (0.99–2.17)	**1.88 (1.25–2.81)**
aOR (95% CI)	-	1.31 (0.97–1.99)	**1.75 (1.11–2.13)**
Vigorous exercise: No (%)	470 (42.9%)	235 (50.2%)	192 (54.4%)
cOR (95% CI)	-	**1.34 (1.08–1.67)**	**1.59 (1.23–2.02)**
aOR (95% CI)		**1.37 (1.07–1.75)**	**1.87 (1.41–2.48)**
Paracetamol use: No (%)	778 (71.3%)	310 (66.5%)	262 (74.6%)
cOR (95% CI)	-	0.80 (063–1.01)	1.18 (0.90–1.56)
aOR (95% CI)	-	0.86 (0.66–1.12)	1.14 (0.84–1.54)

^1^ No-asthma group were free of any asthma responses; ^2^ cOR = Crude Odds Ratio; ^3^ aOR = Adjusted Odds Ratio for other studied personal, environmental, and dietary factors and allergens (95% CI) = 95% Confidence Interval. Bold 95% CIs are statistically significant.

**Table 2:** Dietary factors among school children with diagnosed asthma, children with a 12-month nocturnal cough, and the no-asthma group.

Dietary Factors	No-Asthma Group ^1^ *N* = 1096	Ever (Diagnosed) Asthma *N* = 468	12-Month Nocturnal Cough *N* = 353
Seafoods intake: No (%)	49 (4.5%)	8 (1.7%)	4 (1.1%)
cOR ^2^ (95% CI)	-	**0.37 (0.17–0.79)**	**0.24 (0.09–0.68)**
aOR ^3^ (95% CI)	-	**0.39 (0.17–0.88)**	**0.41 (0.16–0.95)**
Fruit intake: No (%)	507 (46.3%)	197 (42.1%)	138 (39.1%)
cOR (95% CI)	-	0.84 (0.68–1.05)	**0.74 (0.58–0.95)**
aOR (95% CI)	-	**0.72 (0.54–0.96)**	**0.56 (0.40–0.79)**
Vegetable intake: No (%)	485 (44.3%)	218 (46.6%)	171 (48.4%)
cOR (95% CI)	-	1.10 (0.88–1.36)	1.18 (0.93–1.50)
aOR (95% CI)	-	**1.58 (1.18–2.11)**	**2.17 (1.57–3.02)**
Legume consumption: No (%)	188 (17.2%)	105 (22.4%)	64 (18.1%)
cOR (95% CI)	-	**1.40 (1.07–1.82)**	1.07 (0.78–1.46)
aOR (95% CI)	-	1.35 (0.95–1.91)	0.85 (0.57–1.26)
Grain intake: No (%)	794 (72.4%)	344 (73.5%)	268 (75.9%)
cOR (95% CI)	-	1.05 (0.82–1.35)	1.20 (0.91–1.58)
aOR (95% CI)	-	1.04 (0.76–1.41)	1.25 (0.88–1.76)
Nut intake: No (%)	225 (20.5%)	85 (18.3%)	50 (14.3%)
cOR (95% CI)	-	0.87 (0.65–1.14)	**0.64 (0.46–0.90)**
aOR (95% CI)	-	0.71 (0.48–1.03)	0.67 (0.44–1.03)
Dairy product intake: No (%)	599 (54.7%)	226 (48.3%)	156 (44.2%)
cOR (95% CI)	-	**0.77 (0.62–0.96)**	**0.66 (0.51–0.84)**
aOR (95% CI)	-	**0.53 (0.40–0.71)**	**0.38 (0.28–0.53)**
Egg intake: No (%)	338 (30.8%)	185 (39.5%)	132 (37.4%)
cOR (95% CI)	-	**1.46 (1.17–1.84)**	**1.34 (1.04–1.72)**
aOR (95% CI)	-	**1.47 (1.11–1.95)**	**1.60 (1.17–2.19)**
Fast Food consumption: No (%)	156 (14.2%)	67 (14.3%)	45 (12.7%)
cOR (95% CI)	-	1.01 (0.74–1.37)	0.88 (0.62–1.25)
aOR (95% CI)	-	0.92 (0.64–1.33)	0.73 (0.47–1.12)
Meat consumption: No (%)	563 (51.40%)	237 (50.6%)	191 (54.1%)
cOR (95% CI)	-	0.97 (0.78–1.21)	1.11 (0.88–1.42)
aOR (95% CI)	-	0.85 (0.65–1.10)	1.21 (0.90–1.62)

^1^ No-asthma group were free of any asthma responses; ^2^ cOR = Crude Odds Ratio; ^3^ aOR = Adjusted Odds Ratio for other studied personal, environmental, and dietary factors and allergens (95% CI) = 95% Confidence Interval. Bold 95% CIs are statistically significant.

**Table 3:** Allergen sensitizations factors among school children with diagnosed asthma, children with a 12-month nocturnal cough, and the no-asthma group.

Allergens	No-Asthma Group ^1^ *N* = 1096	Ever (Diagnosed) Asthma *N* = 468	12-Month Nocturnal Cough *N* = 353
Indoor Allergens			
*D. pteronyssinus*: No (%)	79 (7.2%)	20 (4.3%)	16 (4.5%)
cOR ^2^ (95% CI)	-	**0.57 (0.35–0.95)**	0.61 (0.35–1.06)
aOR ^3^ (95% CI)	-	0.87 (0.46–1.63)	1.20 (0.57–2.55)
*D. farina*: No (%)	50 (4.6%)	16 (3.4%)	20 (5.7%)
cOR (95% CI)	-	0.74 (0.42–1.31)	1.25 (0.73–2.14)
aOR (95% CI)	-	0.52 (0.23–1.19)	0.86 (0.37–1.96)
Dog hair: No (%)	7 (0.6%)	12 (2.6%)	7 (2.0%)
cOR (95% CI)	-	**4.09 (1.60–10.46)**	**3.14 (1.10–9.04)**
aOR (95% CI)	-	**6.38 (1.80–22.53)**	1.40 (0.39–4.94)
Cat fur: No (%)	176 (16.1%)	109 (23.3%)	76 (21.5%)
cOR (95% CI)	-	**1.58 (1.21–2.07)**	**1.43 (1.06–1.93)**
aOR (95% CI)	-	1.21 (0.88–1.67)	0.81 (0.55–1.19)
Horse hair: No (%)	47 (4.3%)	19 (4.1%)	25 (7.1%)
cOR (95% CI)	-	0.94 (0.54–1.62)	**1.70 (1.03–2.80)**
aOR (95% CI)	-	0.89 (0.48–1.64)	1.31 (0.72–2.39)
*Cladosporium*: No (%)	64 (5.8%)	41 (8.8%)	25 (7.1%)
cOR (95% CI)	-	**1.55 (1.03–2.33)**	1.23 (0.76–1.98)
aOR (95% CI)	-	**1.72 (1.06–2.78)**	1.25 (0.71–2.21)
Outdoor Allergens			
Mugwort (*Artimisia*): No (%)	3 (0.3%)	3 (0.6%)	3 (0.8%)
cOR (95% CI)	-	2.35 (0.47–11.68)	3.20 (0.63–15.42)
aOR (95% CI)	-	-	-
*Chenopodium album*	8 (0.7%)	7 (1.5%)	0 (0.0%)
cOR (95% CI)	-	2.06 (0.74–5.72)	-
aOR (95% CI)	-	1.18 (0.26–5.32)	-
Pigweed (*Amaranthus retroflexus*): No (%)	8 (0.7%)	15 (3.2%)	7 (2.0%)
cOR (95% CI)	-	**4.50 (1.89–10.69)**	2.75 (0.99–7.64)
aOR (95% CI)	-	**3.18 (1.03–9.83)**	**7.76 (1.79–33.56)**
Timothy grass (*Phleum pretense*): No (%)	71 (6.5%)	24 (5.1%)	37 (10.5%)
cOR (95% CI)	-	0.78 (0.48–1.25)	**1.69 (1.11–2.56)**
aOR (95% CI)	-	**0.39 (0.21–0.71)**	0.76 (0.44–1.32)
Bermuda grass (*Cynodon dactylon*): No (%)	158 (14.4%)	115 (24.6%)	98 (27.8%)
cOR (95% CI)	-	**1.93 (1.477–2.53)**	**2.28 (1.71–3.04)**
aOR (95% CI)	-	**1.66 (1.17–2.35)**	**2.55 (1.71–3.79)**
Ragweed (*Ambrosia*): No (%)	25 (2.3%)	19 (4.1%)	19 (5.4%)
cOR (95% CI)	-	1.813 (0.99–3.32)	**2.44 (1.32–4.48)**
aOR (95% CI)	-	1.32 (0.63–2.77)	1.42 (0.68–2.98)
*Candida albicans*: No (%)	27 (2.5%)	0.00%	4 (1.1%)

^1^ No-asthma group were free of any asthma responses; ^2^ cOR = Crude Odds Ratio; ^3^ aOR = Adjusted Odds Ratio for other studied personal, environmental, and dietary factors and allergens (95% CI) = 95% Confidence Interval. Bold 95% CIs are statistically significant.
